# Effect of Influenza Vaccination Inducing Antibody Mediated Rejection in Solid Organ Transplant Recipients

**DOI:** 10.3389/fimmu.2020.01917

**Published:** 2020-10-06

**Authors:** Elisa Cordero, Angel Bulnes-Ramos, Manuela Aguilar-Guisado, Francisca González Escribano, Israel Olivas, Julián Torre-Cisneros, Joan Gavaldá, Teresa Aydillo, Asunción Moreno, Miguel Montejo, María Carmen Fariñas, Jordi Carratalá, Patricia Muñoz, Marino Blanes, Jesús Fortún, Alejandro Suárez-Benjumea, Francisco López-Medrano, Cristina Roca, Rosario Lara, Pilar Pérez-Romero

**Affiliations:** ^1^Instituto de Biomedicina de Sevilla (IBIS), University Hospital Virgen del Rocío, CSIC, University of Seville, Seville, Spain; ^2^Department of Medicine, University of Seville, Seville, Spain; ^3^Servicio de Inmunología, Instituto de Biomedicina de Sevilla (IBIS), University Hospital Virgen del Rocío, CSIC, University of Seville, Seville, Spain; ^4^Reina Sofia University Hospital, Maimonides Institute for Biomedical Research (IMIBIC), University of Córdoba (UCO), Córdoba, Spain; ^5^Vall d’Hebron University Hospital, VHIR, Barcelona, Spain; ^6^University Hospital Clinic, Barcelona, Spain; ^7^Cruces University Hospital, Bilbao, Spain; ^8^University Hospital Marqués de Valdecilla, Santander, Spain; ^9^Belltvitge University Hospital, IDIBELL, University of Barcelona, Barcelona, Spain; ^10^Department of Clinical Microbiology and Infectious Diseases, Hospital General Universitario Gregorio Marañón, Madrid, Spain; ^11^Instituto de Investigaciónn Biomédica Gregorio Marañón, Madrid, Spain; ^12^Department of Medicine, Universidad Complutense de Madrid, Madrid, Spain; ^13^CIBERES (CB06/06/0058), Madrid, Spain; ^14^University Hospital La Fe, Valencia, Spain; ^15^University Hospital Ramón y Cajal, Madrid, Spain; ^16^University Hospital Virgen Macarena, Seville, Spain; ^17^Unit of Infectious Diseases, University Hospital 12 de Octubre, Madrid, Spain; ^18^Instituto de Investigación Biomédica imas12, Madrid, Spain; ^19^Department of Medicine, School of Medicine, Universidad Complutense de Madrid, Madrid, Spain; ^20^National Centre for Microbiology, Instituto de Salud Carlos III, Madrid, Spain

**Keywords:** cytomegalovirus, alloreactivity, donor specific antibodies, anti-human leukocyte antigen, organ rejection

## Abstract

**Introduction:**

Our goal was to study whether influenza vaccination induced antibody mediated rejection in a large cohort of solid organ transplant recipients (SOTR).

**Methods:**

Serum anti-Human Leukocyte Antigen (HLA) antibodies were determined using class I and class II antibody-coated latex beads (FlowPRA^TM^ Screening Test) by flow cytometry. Anti-HLA antibody specificity was determined using the single-antigen bead flow cytometry (SAFC) assay and assignation of donor specific antibodies (DSA) was performed by virtual-crossmatch.

**Results:**

We studied a cohort of 490 SOTR that received an influenza vaccination from 2009 to 2013: 110 (22.4%) received the pandemic adjuvanted vaccine, 59 (12%) within the first 6 months post-transplantation, 185 (37.7%) more than 6 months after transplantation and 136 (27.7%) received two vaccination doses. Overall, no differences of anti-HLA antibodies were found after immunization in patients that received the adjuvanted vaccine, within the first 6 months post-transplantation, or based on the type of organ transplanted. However, the second immunization dose increased the percentage of patients positive for anti-HLA class I significantly compared with patients with one dose (14.6% vs. 3.8%; *P* = 0.003). Patients with pre-existing antibodies before vaccination (15.7% for anti-HLA class I and 15.9% for class II) did not increase reactivity after immunization. A group of 75 (14.4%) patients developed *de novo* anti-HLA antibodies, however, only 5 (1.02%) of them were DSA, and none experienced allograft rejection. Only two (0.4%) patients were diagnosed with graft rejection with favorable outcomes and neither of them developed DSA.

**Conclusion:**

Our results suggest that influenza vaccination is not associated with graft rejection in this cohort of SOTR.

## Introduction

Annual influenza vaccination is the most efficacious method in reducing influenza complications inducing an optimal antibody response in solid organ transplant recipients (SOTR) (1–7), although with lower titers than those in the general population (8). However, a percentage of SOTR, ranging from 1 to 47%, did not respond adequately to influenza vaccine immunization, either with an insufficient antibody response to vaccination or from developing influenza infection after vaccination (9, 10). Different strategies can be used to increase vaccination efficacy such as the use of an adjuvanted vaccine that has been demonstrated to be safe inducing a protective immune response in SOTR (11, 12). Our group demonstrated that having detectable antibody titers at vaccination improves the response to vaccination in SOTR supporting the administration of a booster dose of the influenza vaccine (13, 14), that was later demonstrated to be safe and effective inducing an increased antibody response in a clinical trial (15).

Despite the demonstrated efficacy of the influenza vaccination in SOTR, different authors have suggested that exposure to influenza antigens through vaccination could directly activate alloreactive T and B cells (activating heterologous innate immunity) (12, 13). Influenza vaccination can induce antibody mediated rejection by increasing donor specific anti-human leukocyte antigen (HLA) antibodies (DSA) (16–20). Recognition of donor HLA in the graft by recipient’s T cells can stimulate allograft rejection by the activation of a pro-inflammatory response that includes the activation of B cells producing DSA that can be associated with the development of chronic allograft injury (21, 22). In addition, using strategies to increase vaccination efficacy (high-dose vaccines, booster doses, and adjuvanted vaccines) may contribute to increased allograft rejection and graft dysfunction.

However, the available results concerning this issue are scarce. Danziger-Isakov et al. in 2010 observed an increase in cellular alloimmunity that was not translated into rejection in a study including 17 transplant recipients (23). Similarly, a patient awaiting kidney transplantation was documented with a specific alloreactive kidney cell-specific response after a live attenuated varicella vaccination (24). Regarding the humoral response, in a study including 42 kidney transplant recipients that received a single dose of a pandemic H1N1 ASO3 adjuvanted vaccine, it was reported that *de novo* DSA and non-DSA anti-HLA antibodies developed in five cases (11.9%), not linked to acute rejections (25). In another study including two cohorts of 92 and 59 kidney transplant recipients that received one dose of the seasonal influenza and two doses of the AS03-adjuvanted H1N1 vaccine, respectively, the incidence of *de novo* anti-HLA antibodies was very similar: 17.3 and 11.9%. However, two of these patients (10%) presented thrombotic microangiopathy and humoral rejection, within the next 6 months after vaccination, partly attributed to the development of anti-HLA antibodies (26). However, no events of rejection or *de novo* anti-HLA antibodies were observed in a study including 66 kidney transplant recipients that received trivalent influenza vaccines (27). A recent study has shown no cross-reactivity between HLA class I and II and several virus-specific human monoclonal antibodies, suggesting that the emergence of anti-HLA antibodies in the setting of viral infections or vaccination may be due to bystander activation or dormant HLA specific B cells (28). Our group has demonstrated that administering the influenza vaccine 1 month post-transplantation is safe and efficacious (14), reporting no episodes of rejection, however, we did not address the *de novo* synthesis of DSA.

Based on these results, it would be necessary to conduct large cohort studies in order to explore and clarify the safety of vaccination and its relationship with the induction of DSA and allograft rejection in SOTR. Thus, the aim of this study was to determine whether influenza vaccination induces antibody mediated rejection in SOTR.

## Materials and Methods

### Patient Inclusion

A multicenter, prospective, longitudinal study of consecutive SOTR receiving the influenza vaccines during four consecutive influenza seasons: 2009–2010, 2010–2011, 2011–2012, and 2012–2013 was carried out. Patients were included from the following 12 Spanish hospitals. Virgen del Rocío (Seville), Reina Sofía (Córdoba), 12 de Octubre (Madrid), Gregorio Marañón (Madrid), Ramón y Cajal (Madrid), Vall d’Hebron (Barcelona), Bellvitge University (Barcelona), Clinic Hospital (Barcelona), Cruces (San Vicente de Barakaldo), La Fe (Valencia), Virgen Macarena (Seville), and Marqués de Valdecilla (Santander), all participating in the Spanish Network for the Research in Infectious Diseases (REIPI).

Patients were excluded if they were less than 16 years old, received the transplant less than 1 month ago, had allergies to any of the vaccine components, developed acute rejection 15 days before vaccination, or were pregnant. In patients vaccinated from 2009 to 2012, serum samples were collected at vaccination (baseline) and 5 weeks later. Patients from 2012 to 2013 season came from the TRANSGRIPE clinical trial (15) that randomly received either one or two doses of the vaccine 5 weeks apart. In both arms, serum samples were collected at baseline, 5 weeks, 10 weeks, and 1 year after vaccination. Patients were followed-up for 10 months to record possible adverse events, including events of influenza disease.

### Vaccines

Patients from 2009 to 2010 season received the 2009 (H1N1)pdm monovalent MF59-adjuvanted vaccine containing the strain: A/California/7/2009-H1N1 (Focetria, Novartis, Siena, Italy). Patients from 2010 to 2011 and 2011 to 2012 seasons received the trivalent non-adjuvanted inactivated vaccine (Gripavac, Sanofi-Pasteur MSD, Madrid, Spain) containing the strains: A/California/7/2009-H1N1, A/Perth/16/2009-H3N2, and B/Brisbane/60/2008. Patients from 2012 to 2013 season were randomly assigned to received one or two doses of the trivalent non-adjuvanted inactivated vaccine (Mutagrip, Sanofi-Pasteur MSD) with the strains: A/California/7/2009-H1N1, A/Victoria/361/2011-H3N2, and B/Wisconsin/1/2010.

### Clinical Parameters and Definitions

Baseline characteristics, immunological and clinical response, and adverse effects, including graft rejection and mortality, were recorded. Follow-up clinical parameters were collected using a standardized questionnaire. Graft rejection was confirmed histologically if biochemical or functional abnormalities were detected. Vaccinated patients included all individuals who had received the seasonal influenza vaccine in the previous year or any vaccine in the previous month. Rejection was defined using the Banff criteria (29). Adverse events were assessed according to established criteria (30).

### HLA Determination

We first carried out a sample screening to determine the presence of anti-HLA antibodies in serum using class I and class II antibody-coated latex beads (FlowPRA^TM^ Screening Test, One Lambda, Inc., Canoga Park, CA, United States) by flow cytometry according to the manufacturer’s instructions. A doubling of the PRA percentage at any time point after vaccination from baseline or a 10% increase was considered a positive alloantibody response, and we used single antigen beads to assess for specificity of the alloantibody for positive responses. On the serum samples in which anti-HLA class I and/or class II antibodies were detected in the screening, the specificity of the anti-HLA antibodies was determined using the single-antigen bead flow cytometry (SAFC) assay (Lifecodes LSA^TM^ class I, LSA1, and/or class II, LSA2, Tepnel). Phycoerythrin-conjugated goat anti-human IgG antibody was used as a secondary antibody. Samples were analyzed on a Life Match fluoroanalyzer using Luminex 100 IS v 2.3 as software for data acquisition and Quicktype for Lifematch as analysis software. Positive and negative control sera and beads were included in each test. For validation, all controls met the manufacturer’s specifications. To determine if an individual bead was recognized by a serum sample, only the Median Fluorescent Intensity (MFI) raw value was considered. A sample was considered positive to a specific bead when the MFI raw value was greater than 1500.

### Assignation of DSA (Virtual Crossmatches): Donor HLA Typing

Samples of all deceased donors were routinely typed before recipient selection in loci HLA-A^∗^, B^∗^, and DRB1^∗^ using the PCR-specific sequence primers (SSPs) system (AllSetTM Gold HLA-A, HLA-B, and HLA-DR Low Resolution SSP kits; Invitrogen Ltd., Paisley, United Kingdom). If antibodies against HLA-C, DQ, or DP were detected by SAFC in a pre-transplant patient serum, the donor HLA genotype was performed in the corresponding locus using a PCR-SSP system (AllSetTM Gold HLA-C and HLA-DQ Low Resolution SSP kits and AllSetTM Gold HLA-DPB1 High resolution SSP kit; Invitrogen Ltd). High resolution typing was performed if it was necessary to establish whether the anti-HLA antibodies were DSA (SSP Gold High resolution kits; Invitrogen Ltd), determining whether SAFC detected anti-HLA antibodies in a particular patient were DSA comparing with donor HLA typing.

### Influenza Virus Microneutralization Assays

Microneutralization assays were performed as previously described (8, 14).

### Statistical Analysis

A descriptive statistical analysis was performed. Continuous variables were expressed as median and interquartile ranges. Rates of positive patients for anti-HLA class I and II were compared using the McNemar or Chi square tests and the Bonferroni correction was applied when appropriate. The multivariate logistic regression analysis of factors potentially associated with anti-HLA response included significant variables in the bivariate analyses and those clinically relevant variables (time from transplantation to influenza vaccination, type of transplant, and the number of doses of influenza vaccine administered). For immunogenicity analysis geometric mean antibody titer and the *T*-Student test were used. Results were analyzed by PASW Statistic 18.0.1 software. Statistical significance was established as a *p*-value <0.05.

## Results

### Patient Cohort

A total of 490 SOTR were included in the study, 110 (22.4%) patients received the 2009 (H1N1)pdm adjuvanted vaccine, and 380 (77.6%) received a non-adjuvanted seasonal influenza vaccine. A subgroup of 266 patients receiving the non-adjuvanted vaccine, were randomized to receive one dose; 130 (48.8%), or two doses; 136 (51.2%). Additionally, of the 490 SOTR, 59 (12%) were immunized within the first 6 months post-transplantation, and 431 (88%) after 6 months. Baseline characteristics and clinical parameters of the cohort are described in Table 1. The median age of the cohort was 56 years (IC range 46–63) and the liver was the most frequent transplanted organ (31.6%). As an immunosuppressive regime, most patients (91%) had calcineurin inhibitors (69.4% tacrolimus and 21.6% cyclosporine) and 74.7% mycophenolate mofetil. Most patients (73.4%) had received the influenza vaccine the previous flu-season.

**TABLE 1:** Patient baseline characteristics and comorbidities.

**Variables**	**Total *n* = 490**
Male- *n* (%)	315 (64.3)
Age (median, IQR)	56.0 (46–63)
**Type of transplant**	
Kidney- *n* (%)	143 (29.2)
Liver- *n* (%)	155 (31.6)
Heart- *n* (%)	95 (19.4)
Lung -*n* (%)	92 (18.8)
Combined-*n* (%)	5 (1.0)
**Immunosuppressive therapy**	
Tacrolimus- *n* (%)	340 (69.4)
Mycophenolate mofetil-*n* (%)	366 (74.7)
Cyclosporine- *n* (%)	106 (21.6)
mTOR inhibitors- *n* (%)	65 (13.3)
**Comorbidities- *n* (%)**	
Chronic liver disease- *n* (%)	47 (9.6)
Diabetes mellitus- *n* (%)	133 (72.9)
Chronic heart disease- *n* (%)	99 (20.2)
Chronic kidney disease- *n* (%)	68 (8.8)
Hypogammaglobulinemia- *n* (%)	20 (4.1)
Median time since transplantation-months (IQR)	36.5 (12.0–90.1)
Previous season influenza vaccination- *n* (%)	360 (73.4)

*Data are presented as *n* (%) unless otherwise indicated, IQR, interquartile range.*

### Anti-HLA Antibody Response

**FIGURE 1:**
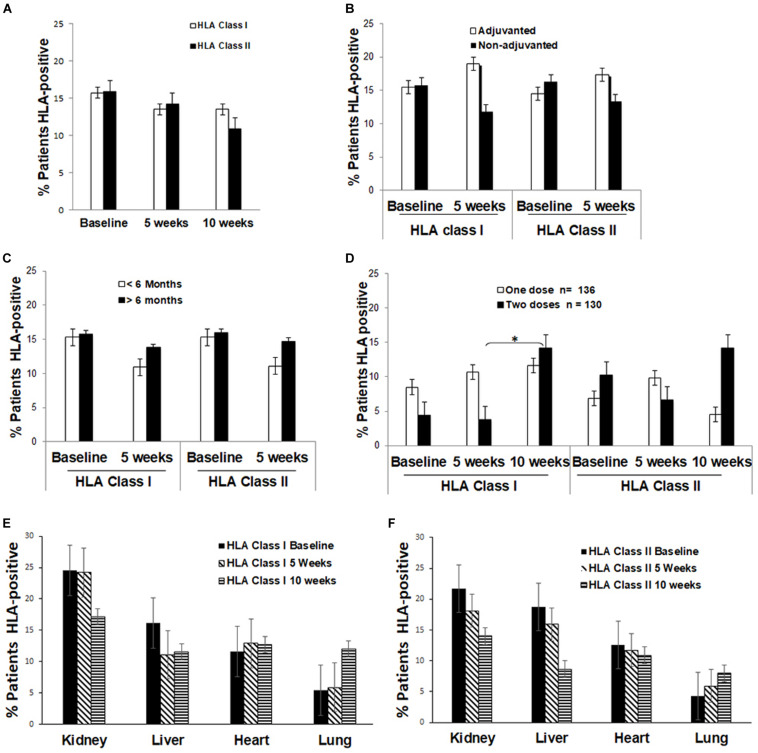
The percentage of patients that were positive for anti-HLA class I or II antibodies: **(A)** Before and after influenza vaccination; **(B)** Comparing patients receiving the adjuvanted or non-adjuvanted vaccine at the indicated time of receiving the influenza vaccine immunization; **(C)** Comparing patients that received the influenza vaccine within the first 6 months (<6 months) or after 6 months (>6 months) post-transplantation; **(D)** Comparing patients receiving one or two doses of the influenza vaccine; **(E)** Comparing patients based on the organ type for anti-HLA class I antibodies; **(F)** Comparing patients based on the organ type for anti-HLA class II antibodies. Notes: Baseline refers to the time of administration of the first dose of influenza vaccine, 5 weeks refers to 5 weeks after the first dose, 10 weeks refers to 5 weeks after the second dose and 1 year refers to 1 year after immunization.*P < 0.05, statistically significant difference.

For all 490 SOTR, anti-HLA antibody response was screened before vaccination (baseline) and 5 weeks after each dose. At baseline, 79 of the patients (15.7%) were positive for anti-HLA class I antibodies and 78 (15.9%) for anti-HLA class II antibodies. None of the patients with pre-existing antibodies had an increase in reactivity after vaccination. The proportion of patients with positive anti-HLA class I and/or class II antibodies did not significantly increase 5 weeks after vaccination (Tables 2, 3 and Figure 1A). The number of patients with positive anti-HLA class I and/or class II did not significantly increase either in the group of patients vaccinated during the first 6 months of the transplant (Tables 2, 3 and Figure 1C) or in those patients who received an adjuvanted vaccine (Table 2, 3 and Figure 1B).

**TABLE 2:** The percentage of patients that were positive for anti-HLA class I antibodies for each of the indicated vaccination strategies.

	**% HLA Class I**					
	N*	**Baseline**	**5 weeks post-vaccination**	N**	**10 weeks post-vaccination**	**1 year post-vaccination**
Total cohort *n*-(%)	490	77 (15.7)	66 (13.4)	260	36 (13.5)	9 (4.9)
**Vaccine composition *n*-(%)**						
Adjuvanted	110	17 (15.4)	21 (19.0)			
Non-adjuvanted	380	60 (15.7)	45 (11.8)			
**Time from Transplant to vaccination *n*-(%)**						
≤6 months	59	9 (15.2)	7 (10.1)			
>6 months	431	68 (15.7)	59 (13.6)			
**Number of vaccine doses *n*-(%)**						
One dose	130	11 (8.0)	14 (10.2)	130	17 (12.5)	4 (2.9)
Two doses	136	6 (4.6)	5 (3.8)	136	19 (14.6)*	5 (3.8)
**Type of transplant *n*-(%)**						
Kidney	153	35 (24.5)	31 (24.4)	64	11 (17.1)	5 (7.8)
Liver	155	25 (16.1)	16 (11.1)	70	8 (11.5)**	1 (1.4)
Heart	95	11 (11.6)	11 (12.9)	55	7 (12.7)	1 (1.8)
Lung	92	5 (5.4)	5 (5.9)	75	9 (12.0)	1 (1.3)
Combined (liver-kidney)	5	1 (20.0)	3 (60.0)	3	1 (33.3)	1 (33.3)

Parameters were compared using the McNemar test or Chi-square test when appropriate. *P = 0.003, **P = 0.043. N*, number of individuals analyzed at baseline and at 5 weeks post-vaccination. N**, number of individuals analyzed at 10 weeks and at 1 year post-vaccination.

**TABLE 3:** Influenza vaccination strategy and anti-HLA class II antibody production.

	**% HLA Class II**					
	N*	**Baseline**	**5 weeks after vaccination**	N**	**10 weeks after vaccination**	**1 year after vaccination**
Total cohort *n*-(%)	490	78 (15.9)	63 (14.0)	260	28 (10.8)	19 (7.1)
**Vaccine composition *n*-(%)**						
Adjuvanted	110	16 (14.5)	19 (17.3)			
Non-adjuvanted	380	62 (16.3)	51 (13.4)			
**Time from Transplant to vaccination *n*-(%)**						
≤6 months	59	9 (15.3)	7 (11.8)			
>6 months	431	69 (16.0)	62 (14.6)			
**Number of vaccine doses *n*-(%)**						
One dose	130	9 (6.6)	13 (9.5)	130	11 (8.0)	10 (7.3)
Two doses	136	13 (10.0)	9 (6.9)	136	17 (13.0)	9 (6.9)
**Type of transplant *n*-(%)**						
Kidney	153	31 (21.7)	23 (18.1)	64	9 (14.0)	9 (14.0)
Liver	155	29 (18.7)	23 (15.9)	70	6 (8.6)	3 (4.3)
Heart	95	12 (12.6)	10 (11.7)	55	6 (10.9)	3 (5.4)
Lung	92	4 (4.3)	5 (5.9)	75	6 (8.0)	3 (4.0)
Combined (liver-kidney)	5	2 (40.0)	2 (40.0)	3	1 (33.3)	1 (33.3)

Parameters were compared using the McNemar test or Chi-square test when appropriate. No differences were observed in all the analyses made. N*, number of individuals analyzed at baseline and at 5 weeks post-vaccination; N**, number of individuals analyzed at 10 weeks and at 1 year post-vaccination.

However, among patients receiving two doses of the trivalent non-adjuvanted inactivated vaccine, the proportion of patients with anti-HLA class I antibodies significantly increased 5 weeks after the second vaccine dose (4.6% vs. 14.6%, *P* = 0.003; Table 2 and Figure 1D), but no increase was observed for anti-HLA class II antibodies. No increase of anti-HLA class I and class II antibodies was noted 1 year after the second vaccination (Tables 2, 3).

The incidence of anti-HLA antibodies stratified by the organ at baseline and during follow-up are detailed in Tables 2, 3 and Figures 1E,F. The levels of anti-HLA antibodies significantly decreased during the follow-up for all patients with class I antibodies at baseline, at 5 weeks, 10 weeks, and 1 year after immunization (54.29% vs. 22.88%, 21.4%, and 8.84%, respectively) while no differences were observed for patients with anti-HLA class II antibodies at baseline.

### Analysis of Patients With Increased Anti-HLA Antibody Response After Vaccination

A total of 75 patients (15.3%) developed *de novo* or had a 10% increase in anti-HLA antibodies after vaccination: 52 patients (69.3%) at 5 weeks, 19 (25.3%) at 10 weeks, and 4 (5.3%) at 1 year. Of them, 41 patients became positive for anti-HLA class I antibodies after vaccination: 11 (26.8%) had received the influenza A/(H1N1)pdm adjuvanted vaccine, 2 (4.8%) were vaccinated within the first 6 months after transplantation, 17 (41.4%) received one dose later than 6 months after the transplant, and 11 (26.8%) received two doses of the vaccine. A group of 25 patients became positive for anti-HLA class II after vaccination: 4 (16.0%) received the pandemic adjuvanted vaccine, 12 (48.0%) received one dose more than 6 months after transplantation, and 9 (36.0%) received two doses. A group of 9 patients became positive for both anti-HLA class I and II antibodies after vaccination: 2 (22.2%) received the pandemic adjuvanted vaccine, 5 (55.5%) two doses, and 2 (22.2%) were vaccinated within the first 6 months post-transplantation. The distribution by organ was: 7 (77.7%) received a liver transplant, 1 (11.1%) kidney, and 1 (11.1%) heart.

In this group of 75 patients that developed anti-HLA antibodies after immunization no significant differences in the levels of MFI were found at 5 weeks after vaccination compared with baseline for anti-HLA class I (2477.78 vs. 2766.69, *P* = 0.647) or class II antibodies (6621.84 vs. 5164.7, *P* = 0.157); at 10 weeks after vaccination for anti-HLA class I (2477.78 vs. 12023.66, *P* = 0.084) and class II (6621.84 vs. 7919, *P* = 0.277) antibodies, and at 1 year after vaccination for anti-HLA antibodies (9510.66 vs. 16609.50, *p* = 0.378).

### Correlation Between Neutralizing Antibody Levels and Development of Anti-HLA Antibodies

No correlation was found between the immunological efficacy (measured by GMT after the immunization) of the influenza vaccine and developing *de novo* anti-HLA antibodies (Table 4).

**TABLE 4:** Geometric mean titer (GMT) after vaccination and anti-HLA class I and II antibodies after vaccination.

	**Positive for anti-HLA class I post-vaccination**			**Positive for anti-HLA class II post-vaccination**			**Positive for anti-HLA class I and II post-vaccination**		
	
**GMT-post vaccination (IQR)**	**Yes**	**No**	***P***	**Yes**	**No**	***P***	**Yes**	**No**	***P***
A/H1N1-pdm	76.13 (20.0–1280.0)	88.24 (5.0–1280)	0.523	40.0 (8.40–160)	90.18 (20–320)	0.675	69.44 (14.1–452.52)	88.82 (20.0–1280.0)	0.446
A/H3N2	83.93 (33.63–1280.0	90.46 (20.0–640.0)	0.933	52.78 (5.0–2228.6)	92.10 (20–640)	0.316	40.0 (14.14–160.0)	92.28 (20.0–640.0)	0.282
Influenza B	195.04 (80.0–1280.0)	146.76 (40.0–1280.0)	0.645	226.27 (80.0–1194.2)	146.57 (40–1280)	0.183	320.0 (56.56–1810.1)	147.97 (40.0–1280)	0.319

*GMT, geometric mean titer. Parameters were compared using *T*-Student test.*

### Multivariate Analysis

A multivariate logistic regression analysis was performed to evaluate possible confounding factors involved in the production of anti-HLA class-II antibodies 5 weeks after the last dose of influenza vaccine and 1 year after vaccination (Table 5). Having detectable HLA antibodies before vaccination was the only factor significantly associated with an increase of anti-HLA class I antibodies [OR = 6.91 (3.6–13.29), *p* = 0.004] and class II [OR = 237.44 (20.08–2341.47), *p* < 0.001] at 5 weeks after the second vaccine dose. Having anti-HLA antibodies was also related with developing anti-HLA class II antibodies [OR = 48.40 (10.20–229.54), *p* < 0.01] at 1 year after vaccination. However, other variables such as the administration of the adjuvanted influenza vaccine or the administration of a seasonal influenza vaccination within the first months after transplantation, were not related with later development of anti-HLA, class I or class II antibodies. Our results also showed that a second dose of the seasonal influenza vaccine was not related to the later development of anti-HLA antibodies, including class II antibodies, which have been related to graft rejection after transplantation. In our SOTR cohort, receiving two doses of the influenza vaccination did not increased the risk of developing class I and II anti-HLA antibodies 1 year after vaccination.

**TABLE 5:** Regression model of factor influencing positive of anti-HLA class I and II.

**Variables**	**% HLA class I positives**				**% HLA class II positives**			
	**10 weeks after vaccination**		**1 year after vaccination**		**10 weeks after vaccination**		**1 year after vaccination**	
				
	**OR (95% CI)**	***P***	**OR (95% CI)**	***P***	**OR (95% CI)**	***P***	**OR (95% CI)**	***P***
First 6 months from Transplant (yes vs. no)	1.17 (0.30–4.48)	0.812	0.501 (0.054–4.60)	0.541	1.17 (0.16–8.15)	0.873	2.36 (0.58–9.58)	0.229
**Type of Transplant**								
Liver	1		1		1		1	
Kidney	1.19 (0.27–5.15)	0.817	0.39 (0.043–3.55)	0.404	1.87 (0.26–13.29)	0.529	0.90 (0.19–4.15)	0.892
Heart	2.46 (0.76–8.00)	0.133	0.23 (0.026–2.14)	0.200	0.69 (0.07–6.81)	0.757	0.29 (0.054–1.56)	0.151
Lung	4E10 (0.00–)	0.999	21.94 (0.93–515.02)	0.055	2E9 (0.00–)	0.999	2.07 (0.02–161.77)	0.743
Mycophenolate (yes vs. no)	1.31 (0.43–4.00)	0.632	1.54 (0.28–8.36)	0.617	19.48 (0.93–404.82)	0.055	0.79 (0.22–2.82)	0.717
Two doses of vaccination (yes vs. no)	2.43 (0.67–8.75)	0.174	2.36 (0.50–11.06)	0.273	16.16 (0.70–369.04)	0.081	0.79 (0.24–2.59)	0.700
Anti-HLA positive at baseline (yes vs. no)	10.53 (2.07–48.77)	0.004	5.96 (0.91–38.80)	0.062	237.44 (20.08–2341.47)	<0.001	48.40 (10.20–229.54)	<0.010

*CI, confidence interval; OR, odds ratio. Parameters were compared using adjusted logistic regression.*

### Donor Specific Antibody Production

Of the 75 patients that developed anti-HLA antibodies in response to vaccination, only 5 patients had DSA (Table 6). One received a combined liver-kidney transplantation and one dose of the 2009 (H1N1)pdm adjuvanted influenza vaccine 57 months after transplantation and developed DSA class I (subtype A^∗^01:01, 02:02) antibodies 5 weeks after vaccination. The second patient was a kidney recipient who received two doses of the non-adjuvanted influenza vaccine 14 months after transplantation, and developed DSA class II (subtype DRB1^∗^01:03 and DRB3) antibodies 5 weeks after the second immunization. In this case DSA was already present in the patient’s serum at baseline and increased after vaccination.

**TABLE 6:** Clinical parameters of patients developing DSA.

**Months from transplantation**	**Organ**	**HLA**	**Subtype**	**Time from baseline to anti-HLA determination**	**Increase in MFI from baseline (%)**	**Rejection**	**Outcome**
57	Combined Liver-Kidney	Class I	HLA I-A 01:01	5 weeks	−83.1%	No	Favorable
14	Kidney	Class II	HLA II DRB1*01:03	10 weeks	−40.2%	No	Favorable
52	Lung	Class II	HLA II DR52	1 year	37.4%	No	Favorable
49	Lung	Class II	HLA II DQ9, DQ2	1 year	83.55%	No	Favorable
6	Kidney	Class II	HLA DQ7, DQ4	1 year	Undetectable at baseline	No	Favorable

The other three patients developed DSA 1 year after vaccination, one of them was a lung recipient who received two doses of the influenza vaccine 52 months after transplantation and developed DSA class II (subtype DR52) antibodies. A second lung recipient received one dose of the non-adjuvanted vaccine 49 months after transplantation and developed DSA class II (subtype DQ2 and DQ9) antibodies. A third kidney recipient received one dose of the vaccine 6 months after transplantation and developed DSA class II (subtype DQ4 and DQ7) antibodies. None of them had graft rejection during the follow-up.

In one patient the specificity of anti-HLA antibodies was not determined because donor genotyping was not available.

No difference in immune suppression was observed between patients who did and did not develop anti-HLA I and II antibodies.

### Rejection Episodes

Only two (0.4%) patients of the cohort studied were diagnosed with graft rejection during the follow-up. A lung recipient (12 months after transplantation) was diagnosed with acute graft rejection 10 days after receiving one dose of the trivalent influenza vaccine. A decrease in FEV_1_ had been detected before vaccination coinciding with sub-therapeutic levels of tacrolimus. A heart recipient (6 months after transplantation) was diagnosed with acute cellular graft rejection IIIA 70 days after receiving a second dose of non-adjuvanted influenza vaccine. Neither of them developed DSA and no other circumstances indicated the presence of acute rejection. Both patients had favorable outcomes after rejection. No differences in pre- and post-vaccination lymphocyte counts were found (1524.8 ± 808.6/ml vs. 1500.3 ± 753.8/ml, *P* > 0.05). In patients with rejection, lymphocytosis after vaccination was not observed.

## Discussion

The presents study suggest that the clinical effects of allostimulation on graft rejection seems extremely low, supporting the safety of different strategies to optimize influenza vaccination response in SOT recipients. Moreover, we observed that the production of DSA antibodies is not related to time elapsed after a transplant or the use of adjuvant or booster doses of the influenza vaccine.

Antibody-mediated rejection is a major determinant of allograft loss. The development of *de novo* DSA after transplantation has been associated with decreased allograft survival (31, 32). The safety of the influenza vaccination in transplant patients has been questioned since some studies have suggested that antigens included in the influenza vaccine composition may cross-react with alloantigens involved in graft rejection, raising the risk of a vaccination-induced alloimmune response (27, 33). In the present study we have analyzed different strategies of influenza vaccination and their influence on the production of DSA and/or rejection in SOT recipients and evaluated the clinical relevance of DSA.

Higher MFI levels have been associated with poorer outcomes. In our cohort, although the method used to measure anti-HLA antibody levels is semiquantitative, having anti-HLA antibodies before immunization did not predispose patients to an increased risk for developing anti-HLA antibodies after vaccination considering the MFI levels before and after vaccination.

We also found that patients receiving the vaccine within 6 months after transplantation had less of a alloreactive response, which may be related with the higher levels of immunosuppressors during this period.

In the present report, the immune response triggered against influenza antigens in the vaccine composition was not associated with the production of *de novo* DSA or with rejection, with similar outcomes in the different types of transplants studied. Contrary to what has been previously suggested (26), our results show that having an adjuvant in the composition of the vaccine has little or no influence on alloimmune response and graft rejection.

The proportion of graft rejection was low in our series, despite all patients being closely followed up, and biopsies were performed in cases that biochemical or functional abnormalities were detected. However, subclinical rejection could not be ruled out. The low incidence of graft rejection could be also explained by the time elapsed since transplantation, more than 1 year for three-quarters of the patients included.

In general, increased risk of graft rejection and lower vaccine efficiency occurs during the first months of transplantation (4, 34). In a previous report we found a similar response to influenza vaccination in patients vaccinated before or after 6 months post-transplantation (14). In this study, the immunological impact of influenza vaccination, assessed by the production of anti-HLA antibodies and DSA, was evaluated, concluding that the timing of influenza vaccinations after transplantation had no influence on the production of anti-HLA antibodies, DSA, or rejection.

Regarding the risk of an alloimmune response against influenza antigens after the administration of a booster dose, we found that although a second immunization dose was associated with developing more anti-HLA class I antibodies, no differences were found regarding the production of DSA and graft rejection. It is not clear in the literature whether DSA have clinical relevance since most of the results available are retrospective. Although some DSA may be associated with a rapid decline in graft function, it may take months to years before a pathologic correlation is seen in others (35).

Other authors have previously studied the induction of donor-specific HLA antibodies after vaccination using the conjugated Streptococcus pneumonia vaccine. Their results show that no *de novo* HLA antibodies were developed in kidney recipients, however, they suggested that female kidney transplant recipients may be more susceptible to the induction of HLA antibodies after vaccination (36, 37). In addition, a recent meta-analysis suggested no effect on the development of *de novo* donor-specific antibodies and no increases in acute rejection after vaccination in SOT recipients. While most of the papers included studied influenza vaccination, measles, hepatitis B, varicella, tetanus, diphtheria, and polio vaccination have also been assessed (38).

Some authors have proposed that rejection is linked to reaching a threshold of alloreactive memory T cells (33). In line with this idea, we found that patients exposed twice to influenza antigens were more prone to have an anti-HLA class I antibody. However, only one SOTR developed *de novo* anti-HLA DSA antibodies after a second immunization, and this patient had a favorable outcome, with no rejection episodes.

The HLA system is highly polymorphic with numerous alleles. Class II antibodies have been more frequently related to graft rejection, particularly those targeting HLA DQ antigens (39, 40). Some authors suggest that there may be a higher risk of acute and chronic allograft rejection when *de novo* DSA are found on routine monitoring at any time post-transplantation (41). Our results do not support this idea since, only four patients had increased DSA class II levels after influenza vaccination, and none of them had clinical or functional graft rejection. Additionally, only two patients had graft rejection in this cohort and none developed *de novo* DSA after influenza vaccination suggesting that rejection was not related to influenza immunization.

Some limitations might be considered. Firstly, we were unable to establish the timing after immunization for the development of antibodies since no samples were collected between 10 and 56 weeks. Secondly, biopsies were only performed if rejection was suspected in all but heart transplant recipients, who had regular histological studies. In patients who developed DSA, biopsies were not performed and therefore subclinical rejection can not be ruled out. In any case, it had no impact on graft function as observed during follow-up. Thirdly, this paper analyses the production of HLA antibodies and its association with antibody mediated rejection. However, it does not exclude the possibility of vaccine induced T cell allorecognition. Finally, no information regarding other types of vaccines different from influenza or pneumococcus is available which limits the extrapolation of these results to other vaccines.

In summary, our results suggest no association between influenza vaccination and graft rejection in SOTR. In addition, strategies to improve the immune response after vaccination such as earlier vaccination after transplantation, the use of an adjuvant in the composition of the vaccine, or the use of a booster dose are effective and safe and may not increase rates of rejection.

## Data Availability Statement

The datasets generated for this study are available on request to the corresponding author.

## Ethics Statement

The studies involving human participants were reviewed and approved by the Comité Ética de la Investigación del HU Virgen del Rocío de Sevilla. The patients/participants provided their written informed consent to participate in this study.

## Author Contributions

AB-R performed all the experiments, analyzed the results, and generated all the figures and the manuscript. CR, TA, MA-G, and RL collected the clinical data, performed the statistical analysis of the data, and generated the manuscript. MA-G, JT-C, JG, AM, MM, CF, JC, PM, MB, JF, AS-B, FL-M, and CR provided patient care and participated in the discussion, and preparation of the manuscript. FGE and IO performed the DSA procedures and participated in the discussion, and preparation of the manuscript. EC and PP-R designed the research, responsible for the project, and preparation of the manuscript. All authors contributed to the article and approved the submitted version.

## Conflict of Interest

PP-R is a founder and shareholder of Vaxdyn, S.L., a biotechnology company developing vaccines. The remaining authors declare that the research was conducted in the absence of any commercial or financial relationships that could be construed as a potential conflict of interest.
